# Second magnetization peak effect, vortex dynamics, and flux pinning in 112-type superconductor Ca_0.8_La_0.2_Fe_1*−x*_Co_*x*_As_2_

**DOI:** 10.1038/srep22278

**Published:** 2016-03-07

**Authors:** Wei Zhou, Xiangzhuo Xing, Wenjuan Wu, Haijun Zhao, Zhixiang Shi

**Affiliations:** 1 Department of Physics and Key Laboratory of MEMS of the Ministry of Education, Southeast University, Nanjing 211189, China

## Abstract

Investigation of vortex pinning and its relaxation is of great importance for both basic physics and technological applications in the field of superconductivity. We report a great improvement of superconducting properties in the recently discovered 112-type superconductors (Ca, La)FeAs_2_ through Co co-doping. High critical current density *J*_*s*_(5 K) *>* 2^*^10^6^ A/cm^2^ is obtained and pronounced second peak effect is observed in magnetization hysteresis loops. Both the dynamic and static relaxation studies result in comparable and sizable relaxation rates *S* or *Q*, indicating a fast vortex creep. The second magnetization peak (SMP) is found to be strongly associated with a crossover from elastic to plastic vortex creep. Above the crossover, plastic vortex creep governs the vortex dynamics in a wide range of temperatures and fields. A good scaling behavior of the normalized pinning force density *f*_*p*_ by formula *f*_*p*_ = *h*^*p*^(1*−h)*^*q*^ (^*p*^ = 1.44, *q* = 1.66, *h* = 0.44) is revealed, which demonstrates an important contribution from core normal point-like pinning sites. To better understand the SMP phenomenon, we discuss the related physical scenario as well as the affecting factors in the SMP occurrence.

Investigation of vortex physics in type II superconductors has been a subject of intense interests especially after the discovery of high-*T*_*c*_ superconductivity in cuprates^1^. Many fascinating vortex phenomena were observed and thus accelerated the establishment of various theoretical models^2^. Among the complex vortex phenomena, second magnetization peak (SMP, also known as fishtail) effect in the field-dependent magnetization (*MH* curves) measurement, is widely observed in various kinds of type II superconductors, including low-*T*_*c*_ superconductor (LTS) Nb_3_Sn^3^, high-*T*_*c*_ cuprates YBa_2_Cu_3_O_1*−δ*_ and Bi_2_Sr_2_CaCu_2_O_*y*_^4^,^5^, MgB_2_^6^ and the recently discovered high-*T*_*c*_ iron-based superconductors (IBSs)^7^,^8^,^9^,^10^,^11^,^12^,^13^,^14^,^15^.

The occurrence of SMP shows strong system-specific feature. In cuprates, different vortex dynamical mechanisms including crossover from elastic to plastic (E-P) vortex creep^4^, vortex order-disorder phase transition^16^, vortex lattice structural phase transition (VL)^17^, surface barriers^18^, etc., were proposed in SMP interpretation. In iron pnictides, SMP has been observed in all of the four main systems, 1111-type SmFeAs(O, F) and NdFeAs(O, F)^8^,^19^, 122-type (Ba, K)Fe_2_As_2_ and Ba(Fe, Co)_2_As_2_^9^,^12^, 111-type LiFeAs^14^, and 11-type Fe_1 + *x*_(Te, Se)^13^,^15^,^20^,^21^. However, similar as cuprates, different explanations were proposed^7^,^8^,^14^,^19^. A widely applied model is the idea of E-P vortex creep crossover. In 111-type LiFeAs, supported by the strong temperature-dependent peak position *H*_sp_, VL model was applied^14^. In the more anisotropic 1111 system, a three-dimensional (3D) ordered to 2D disordered vortex lattice transition was suggested^8^,^19^. In 122-type Ba(Fe, Co)_2_As_2_, controversial models of both collective to plastic crossover and VL transition were proposed^7^,^22^. Because of the various possibilities, no general consensus or clear understanding has yet been reached about the underlying mechanism in SMP occurrence. In comparison with cuprates, the less anisotropy *γ* and larger coherence length *ξ* in IBSs^23^,^24^,^25^,^26^,^27^, combined with the moderate *T*_*c*_, jointly provide opportunities to explore vortex physics between LTS and high-*T*_*c*_ cuprates. In IBSs, 1111 system possesses the highest *T*_c_ and moderate anisotropy (*γ* ~ 4−9) close to cuprates. Due to the relative difficulties in growing sizable single crystal, few magnetization studies have been reported in 1111 system^28^,^29^. The recently discovered 112-type superconductor (Ca, La)FeAs_2_ bears high *T*_c_ over 40 K and moderate *γ* (2 < *γ* < 4.2) located between the nearly isotropic iron-based 122 system and the 1111 system^24^,^26^,^30^. The intermediate *T*_c_ and moderate anisotropy make the 112-type superconductors to be a unique candidate for link studies of vortex physics between iron pnictides and cuprates. Meanwhile, in comparison with other iron-based systems, the special monoclinic crystal structure and the unique additional arsenic chain in unit cells enable one to explore the influence of interlayer coupling on vortex interaction in layered superconductors. Unfortunately, due to the relatively poor superconductivity (SC) in the previously grown 112-type crystals^31^,^32^,^33^, no detailed magnetization analysis has been performed. Recently, Co doping has been used to improve the superconducting properties of 112-type polycrystals^34^ In this work, we report a large superconducting critical current *J*_s_ over 2*10^6 ^A/cm^2^ in our Co-co-doping 112-type superconductor Ca_0.8_La_0.2_Fe_1−*x*_Co_*x*_As_2_ single crystal. We found a well pronounced SMP effect in the field-dependent magnetization measurement of our sample, which, to our knowledge, has not yet been reported. Results of this work show that E–P vortex crossover is strongly associated to SMP, and the flux pinning is core normal point-like pinning, which arises from point defects that may come from La and Co doping. Detailed affecting factors of SMP occurrence will be discussed later in this paper.

## Results

**Figure 1:**
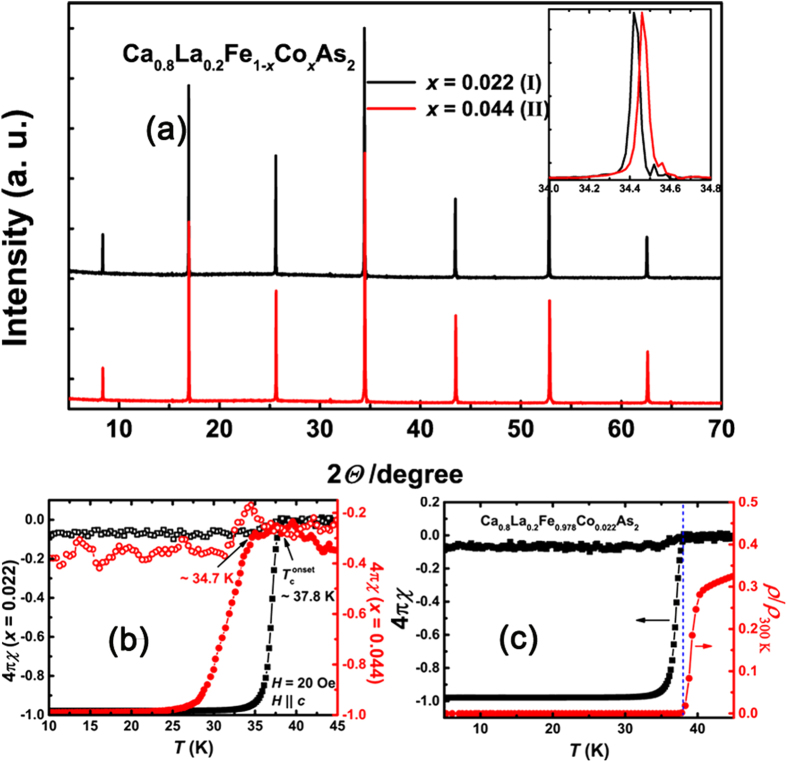
(**a**) XRD patterns for Ca_0.8_La_0.2_Fe_1*−x*_Co_*x*_As_2_ single crystals. Inset: Comparison of the (004) peak between two crystals with different doping levels. (**b**) Temperature dependence of the DC magnetic susceptibility (demagnetization corrected) for sample I (black) and sample II (red). The original magnetization data were collected by zero-field cooling (ZFC, solid symbols) and field cooling (FC, open symbols) processes. (**c**) Temperature dependence of resistivity and SC volume fraction for Ca_0.8_La_0.2_Fe_1*−x*_Co_*x*_As_2_ (*x* = 0.022). Consistent resistive and magnetic transitions can be observed.

The X-ray diffraction (XRD) patterns with only K*α*1 contribution are shown in Fig. 1(a). Only (00*l*) peaks for the monoclinic phase can be observed, indicating good *c*-axis orientation. The full widths at half maximum (FWHM) in both crystals show very small values *∼* 0.05, *i.e.*, a very good crystalline quality is demonstrated. Figure 1(b) shows the temperature dependence of the DC magnetic susceptibility (demagnetization corrected) of the studied crystals. The full diamagnetism in both crystals clearly indicates bulk superconductivity. The sharp superconducting transition in our crystals can hardly be seen in 112-type IBSs^31^,^32^,^33^,^35^,^36^, also manifesting the high sample quality. Figure 1(c) shows the consistent resistive and magnetic superconducting transitions. In case of non-uniform superconductors with broad distributed *T*_c_, the measured transport *T*_c_ is usually higher than that in magnetic measurement for possible existence of high-*T*_c_ thoroughfares. Therefore, on basis of the consistent transport and magnetic *T*_c_ values for the Co-co-doping 112 crystal, we believe our crystal studied in this work is uniform enough and the high-*T*_c_ superconductivity is bulk in nature. The observation of SMP is also directly benefitted from the uniform superconductivity.

**Figure 2:**
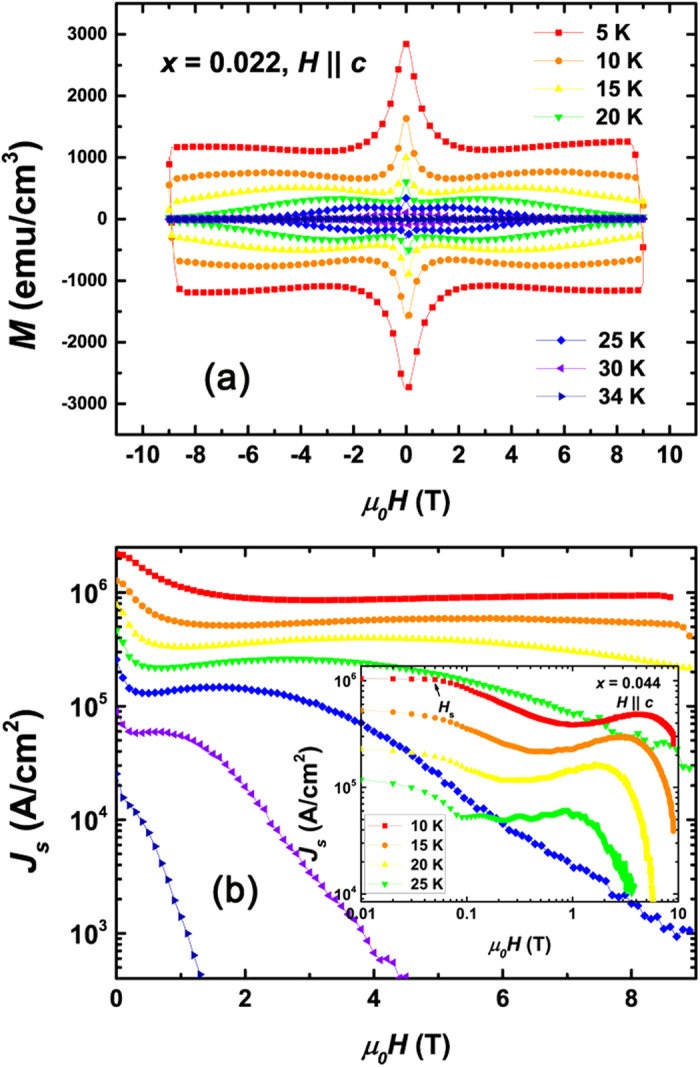
(**a**,**b**) Magnetic field (*H*) dependence of magnetization and critical current density *J*_s_ at various temperatures. *H* is applied along the crystal’s *c*-axis. Large *J*_s_ value over 2*10^6 ^A/cm^2^ was obtained at *T* = 5 K. Inset in (**b**) shows *J*_s_(*H*) for sample Ca_0.8_La_0.2_Fe_1*−x*_Co_*x*_As_2_ (*x* = 0.044) within a log-log plot. *H*_s_ is defined as the upper critical point for single-vortex region in which *J*_s_ is field-independent.

Figure 2(a) shows the typical *MH* curves with SMP effect which was obtained with a constant field sweeping rate d*H* /d*t* = 100 Oe/s for *H* applied along the crystal’s *c*-axis. The symmetric *MH* curves suggest dominant bulk pinning instead of surface barrier, which guarantees the application of Bean critical state model in the critical current density *J*_*s*_ calculation. A pronounced second magnetization peak can be clearly observed. The second peak position *H*_sp_ moves up quickly as temperature decreases. The SMP phenomenon quite resembles that in YBa_2_Cu_3_O_7*−x*_ and 122-type and 11-type IBSs^4^,^10^,^13^,^37^, implying an analogous origin of the peak effect. Using the famous Bean model^38^, *J*_*s*_ = 20*M*_diff_/*a*(1−*a*/3*b*), where *M*_diff_ (emu/cm^3^) is *M*_down_-*M*_up_, *M*_down_ and *M*_up_ are magnetization when sweeping fields down and up, respectively, calculated current density *J*_*s*_ (A/cm^2^) for *H || c* was plotted in Fig. 2(b). Here *a* (cm) and *b* (cm) are sample width and length (*a* < *b*). Remarkably, *J*_*s*_ at 5 K shows large values over 2*10^6 ^A/cm^2^, in sharp contrast to the low values in electron-doped Ca(Fe_1−*x*_Co_*x*_)_2_As_2_^39^ (*J*_s_ ~ 10^4 ^A/cm^2^) and also the Co-free 112-type crystals (*J*_s_ ~ 10^5 ^A/cm^2^)^30^. As can be seen in the inset in Fig. 2(b) and in supplementary information (SI) Fig. S1, *J*_*s*_ changes subtly at low field region up to *H*_s_. Here, the characteristic field *H*_s_ for single-vortex region is defined as the critical point where *J*_*s*_(*H*) change from a constant value to a decreasing function of *H*.

Since the occurrence of SMP is quite advantageous in view of practical application and investigation of its origin is also helpful for understanding the fundamental question of underlying vortex physics, we performed a detailed vortex pinning and relaxation study on this new type superconductor. Magnetization relaxation in superconductors occurs because of the nonequilibrium spatial distribution of vortices which is determined by the competition of the external Lorentz force, the disorder induced pinning force, and the thermal fluctuation. Due to thermal fluctuation, a logarithmic time dependence of magnetization (*M*(*t*)) can often be observed experimentally, which is explained by the proposed linear dependence of the barrier energy *U* (also known as the flux activation energy) on current density *J*, *i.e.*, *U* = *U*_0_(1−*J*/*J*_*c*_) where *U*_0_ is the barrier energy in absence of flux creep^40^. Later, many other models of nonlinear *U*(*J*) dependence were established due to the observation of deviation from the logarithmic *M*(*t*). There is a useful interpolation formula for the barrier energy, which covers the known *U*(*J*) functions^41^,


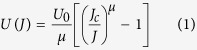


Here, *μ* is the glassy exponent whose value determines the vortex pinning regime. Eq. (1) leads to the time-dependent magnetization and current density of the forms


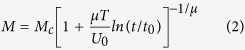


and


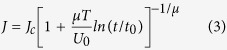


where *M*_c_ (*J*_c_) is the original magnetization (critical current density) before creep and *t*_0_ is the characteristic relaxation time (usually 10^−6^–10^−12 ^s). To make clear the vortex regime, it is important to determine the glassy exponent *μ*.

**Figure 3:**
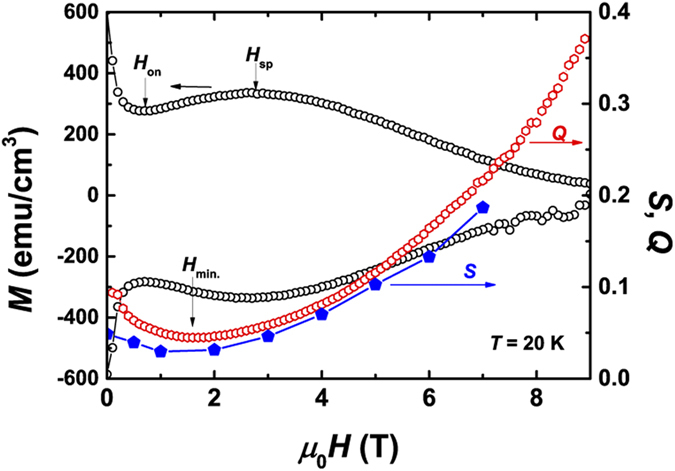
Field dependence of magnetization *M* and magnetization-relaxation rates *S* and *Q*.

To check the influence of the field sweeping rate *dH*/*dt* on the SMP, the dynamical relaxation study was also performed by measuring *MH* curves with different *dH*/*dt* (see SI, Fig. S3(a)). We have compared the magnetization difference (Δ*M*) at *H*_on_ and *H*_sp_ for each *MH* curve with different *dH*/*dt* (see SI, Fig. S3(b)). The value of Δ*M* is found to slightly increase with increasing *dH*/*dt*, which suggests that SMP will persist for higher *dH*/*dt*. Figure 3 shows the field dependence of static (dynamic) relaxation rate *S* (*Q*) together with the *MH* curve. *S* and *Q* values are obtained via definitions *S* ≡ *d*(ln *M*)/d(ln *t*) and *Q* ≡ *d*(ln *M*)/*d*(ln *dB*/*dt*), respectively. Apparently, *S* and *Q* show very similar values and trends with field, indicating the compatibility of the static and dynamic relaxation methods. The large relaxation rates (*S* or *Q* > 0.03) are very similar to those obtained in 1111-type, 122-type, and 11-type IBSs^21^,^28^,^42^. Additionally, a minimum value is noticed in *S*(*H*) (or *Q*(*H*)) curve. The field position *H*_min._ of the minimum *S* (or *Q*) just locates between the onset field *H*_on_ and the peak position *H*_sp_ of SMP. As will be shown later in *H*–*T* phase diagram, *H*_min._ is very close to the characteristic field separating elastic-dominated and plastic-dominated vortex creep regimes.

**Figure 4:**
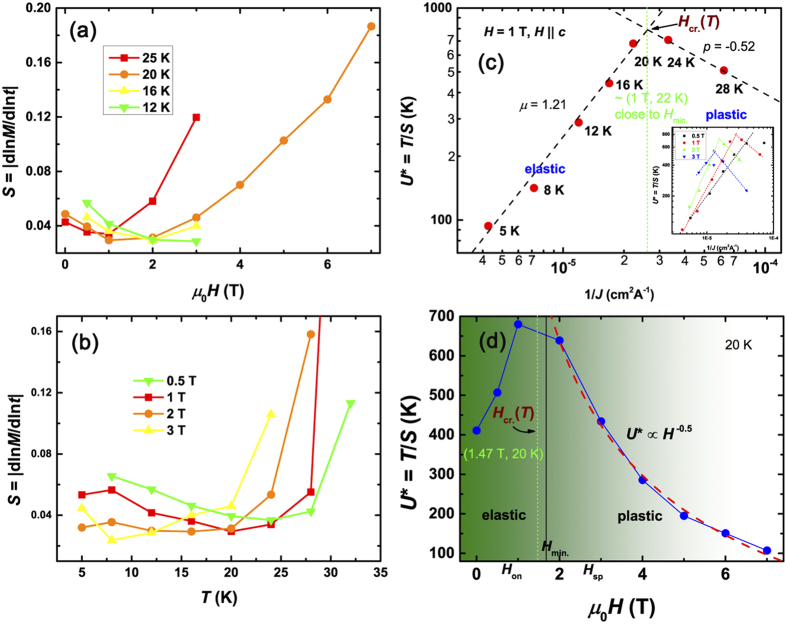
(**a**,**b**) Field and temperature dependence of relaxation rate *S*. (**c**,**d**) Inverse current-density and field dependence of effective pinning energy U^*^. The red dash line in (**d**) is a fitting curve based on 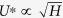. The green dash lines in (**c**,**d**) are guide lines to the cross point between the two different U^*^(1/*J*) (or U^*^(*H*)) dependencies. *H*_cr._(*T*) determined by the field and temperature position of the green dash lines is the characteristic field for E-P crossover at certain temperature. Inset in the bottom right corner in (**c**) shows the U^*^(1/*J*) relations for other fields. The dash lines are guides for the crossover field *H*_cr._(*T*) between different U^*^(1/*J*) dependencies.

Figure 4(a,b) show the temperature and field dependence of relaxation rate *S*. Note that, a remarkable result extracted from interpolation formula is the successful prediction of plateau with theoretical *S* value of 0.02–0.04 in the intermediate temperature range within the collective creep model for *U*_0_ ≪ *μT*ln(*t/t*_0_)^43^. The comparable *S* values and plateau were observed in many other superconductors including Y-Ba-Cu-O^43^, 122-type^44^, 11-type IBSs^21^, and also the present 112-type superconductor, which proves the validity of application of the weak collective pinning theory. By defining the effective pinning barrier U^*^ = *T/S*, one can get U^*^ = *U*_0_ + *μT*ln(*t/t*_0_). Combined with Eq. (3), U^*^ can be represented by U^*^ = *U*_0_(*J*_*c*_*/J*)^*μ*^. Therefore, the glassy exponent *μ* can be easily obtained by the slope in the double logarithmic plot of U^*^ vs 1/*J* (see Fig. 4(c)). In low temperature region (large *J*), the obtained *μ* (*∼*1.21) value resides between that of single-vortex (*μ* = 1/7) (or intermediate-bundle) and small-bundle regimes, indicating contributions from different pinning types. The *μ* value in our experiment is close to many other IBSs^12^,^20^,^21^,^42^,^44^. In contrast, in high temperature (low *J*) region, the obtained slope is ~ *−*0.52, which is in good agreement with predicted *p* = −0.5 in plastic-creep theory^4^. From another point of view, under the model of E-P crossover, the pinning barrier *U* will show different field dependencies^4^. In elastic creep regime, the barrier potential U^*^ ∝ *H*^*ν*^*J*^*−μ*^, where *ν* is a positive number. U^*^ increases with field. On the other hand, in plastic creep regime, similar to diffusion of dislocations in atomic solids, U^*^ (≃*U*_0_) is proportional to 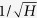, decreasing with field. As can be seen in Fig. 4(d), the above two laws were strictly followed for our studied crystal. Therefore, the U^*^(1*/J*) and U^*^(*H*) behaviors in Fig. 4(c,d) clearly indicate a crossover from elastic to plastic vortex creep regimes. In Fig. 4(c,d), the characteristic field *H*_cr._(*T*) determined by the cross point between different U^*^(1*/J*) (or U^*^(*H*)) relations (indicated by green dash lines) is the critical point separating elastic-dominated and plastic-dominated regimes.

**Figure 5:**
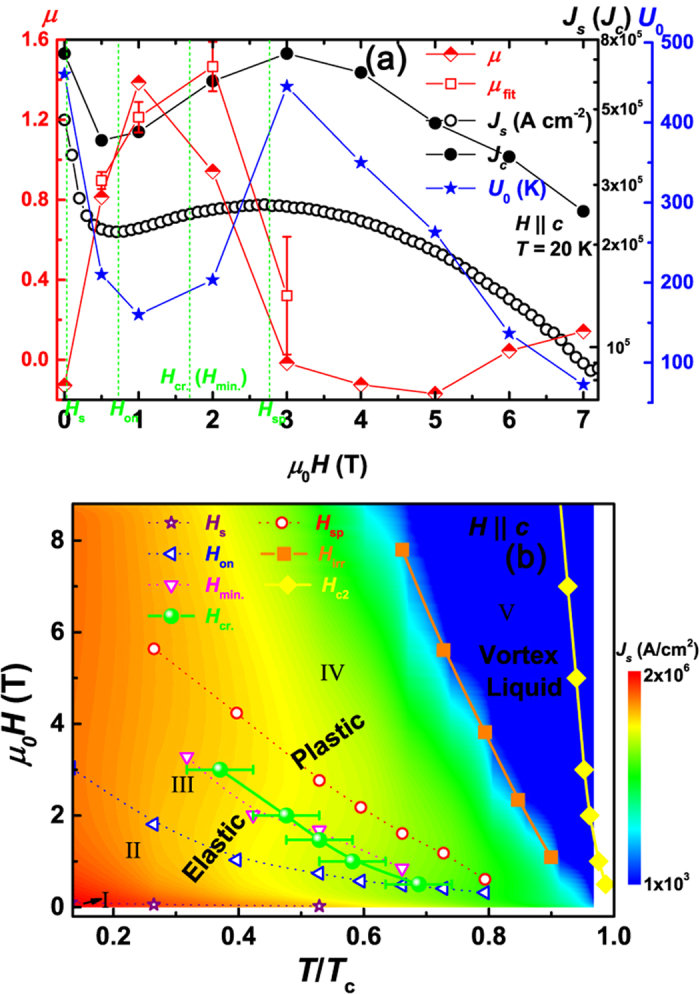
(**a**) Field dependence of critical current density *J*_s_ (*J*_c_), glassy exponent *μ*, and barrier energy *U*_0_. *μ* and *U*_0_ are directly obtained through Eq. (2) fitting of the original measured *M*(*t* *>* 50 s) data. *J*_c_ is calculated from *M*_c_ obtained in Eq. (2) fitting. *μ*_fit_ is obtained from the method in Fig. 4(c). The characteristic fields were also noted in the figure. (**b**) Vortex phase diagram of Ca_0.8_La_0.2_Fe_0.978_Co_0.022_As_2_ with various characteristic *H*(*T*). The color contour represents the critical current density *J*_s_ as functions of temperature and field. *H*_c2_ values are obtained from temperature-dependent resistivity curves under different magnetic fields using the criterion of 90% *ρ*_*n*_ (data shown in SI, Fig. S5). The irreversibility field *H*_irr_ is obtained by extrapolating *J*_s_ to zero in the *J*_s_^1/2^ vs. *H* curves. The solid and dash lines are guide to the eye.

An alternative more direct way to obtain *μ* and barrier energy is through Eq. (2) fitting. To exclude the influence of field overshoot, relaxation data for *t* < 50 s is ignored. In Fig. 5(a), the field-dependent *μ*, *J*_s_, *J*_c_, and *U*_0_ at *T* = 20 K were plotted together. The values of *μ*, *J*_c_ (calculated from *M*_c_), and *U*_0_ are obtained from Eq. (2) fitting. In order to check the possible errors arising from the large uncertainty of the 4-parameter (Eq. (2)) fitting, *μ*_fit_ obtained from the collective pinning model (see Fig. 4(c)) is also added in Fig. 5(a) for comparisons. As can be seen, the *μ* values obtained by two different ways nicely coincide with each other, which ensures our further investigation of vortex regimes from the evolution of *μ* vs. *H*. Apparently, there are complex creep regime changes at low field region up to 3 T. For the convenience of comparison, the positions of some characteristic fields are also noted in Fig. 5(a). For *H* = 0, *μ* value is just below 0 which is a clear signature of single vortex creep. As *H* increases below *H*_cr._ (*H*_min._), *μ* increases fast to positive values larger than 1, indicating the dominant role of weak collective pinning. The *μ* values reside between that for single-vortex (~1/7) and small bundle (~3/2) vortex creep. For *H* ≥ *H*_cr._ (*H*_min._), *μ* value drops quickly to a negative value which is inconsistent with the collective creep theory. Note that, it is inconceivable to suppose a crossover to single-vortex regime at a field higher than the field where single-vortex to collective vortex crossover takes place. As pointed out in Fig. 4(d), the field dependence of U^*^ (∝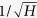) for *H* ≥ *H*_cr._ (*H*_min._) shows quite good agreement with the dislocation mediated mechanism of plastic vortex creep. Therefore, the negative *μ* values indicate plastic vortex dominated creep. It is worth noting here, *μ* at *H*_sp_ has already dropped to very small values, which means dominant plastic regime appears before *H*_sp_. The evolution of *μ*(*H*) completely resembles that in YBa_2_Cu_3_O_1−*x*_, in which SMP origin was ascribed to E–P crossover^4^. Based on the above analysis, the vortex phase diagram is established (see Fig. 5(b)). The whole *H*-*T* phase diagram of vortex lattice is obtained by adding the irreversibility field *H*_irr_ whose value is determined by extrapolating *J*_s_ to zero in the *J*_s_^1/2^ vs. *H*. The details of different regions in phase diagram will be discussed later.

**Figure 6:**
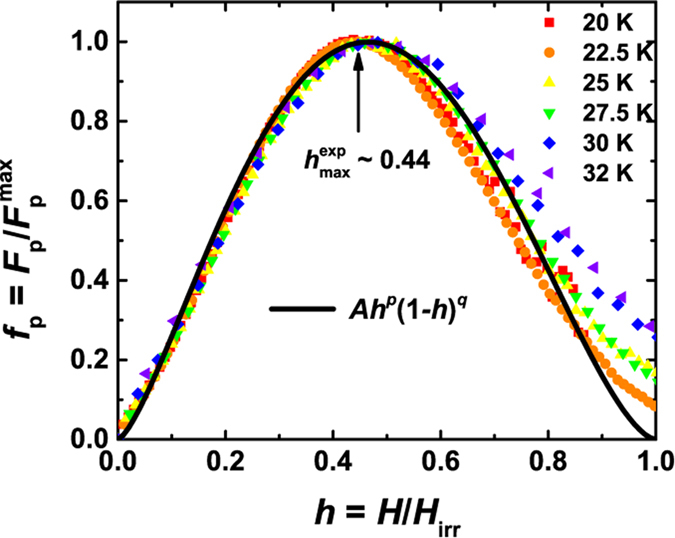
The scaling of the normalized pinning force density *f*_*p*_ for different temperatures as a function of the reduced field *h* based on the *f*_p_(*h*) scaling function. The data measured at different temperatures can be well scaled together with a maximum field *h*exp max *∼* 0.44.

At last, to gain more insight to the underlying pinning mechanism in 112-type superconductor, we investigated the field dependence of the pinning force density *F*_*p*_ = *μ*_0_*HJ*_*c*_. According to Dew-Hughes model^45^, the reduced field *h* (usually *h* = *H*/*H*_irr_) dependence of the normalized pinning force density *f*_*p*_ measured at different temperatures should fall onto one curve in case of existence of a single dominant pinning mechanism. The scaling function is 
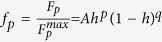
, where *A* is a constant, and *p* and *q* are two parameters giving information of the pinning mechanism. In Fig. 6, *f*_*p*_(*h*) at different temperature are shown and found to be well scaled. The fitting based on the scaling function leads to *p* = 1.44 and *q* = 1.66. The obtained 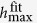 = *p/*(*p* + *q*) = 0.46 is consistent with the experimental value 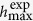 (~0.44). Comparing with these parameters for core normal point-like pinning within Dew-Hughes model (*p* = 1, *q* = 2, and 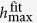 = *p/*(*p* + *q*) = 0.33), small deviations exist. The obtained *h* value is very close to that in Ba_0.6_K_0.4_Fe_2_As_2_ (*h* ~ 0.43) and BaFe_1.8_Co_0.2_As_2_ (*h* ~ 0.45)^11^,^46^. In BaFe_1.8_Co_0.2_As_2_, such *h* value was considered to be an indication of dense vortex pinning nanostructures, which may be resulted from cobalt ions. It was also suggested that such pinning centers are probably weak pinning sites^11^. Such scene can also be applied in the present material, *i.e.*, pinning sites in our sample are also arising from random distributed nano-scale point-like defects, which was commonly found in the previous studies of IBSs^10^,^13^,^42^,^47^.

## Discussions

On basis of the above magnetization relaxation study in Ca_0.8_La_0.2_Fe_1−*x*_Co_*x*_As_2_, we classified five different regions in the *H*–*T* phase diagram (see Fig. 5(b)). And all the pinning centers were divided into two groups: the sparse strong pinning centers and the dense weak pinning centers. For these in the first group, their sizes are larger than the superconducting coherence length, while for these in the second group, their sizes are smaller. For 0 < *H*(*T*) < *H*_s_(*T*) in region I in the phase diagram, vortices are well separated and act like non-interacting objects. Almost all of them are pinned by strong pinning centers (single-vortex creep), which results in a large superconducting critical current density *J*_s_ and a large pinning energy *U*_0_ as well. At higher magnetic fields (region II from *H*_s_(*T*) to *H*_on_(*T*)), additional vortices are introduced. As a result, when the magnetic field is increasing, the number of vortices becomes larger than that of strong pinning centers, and those extra vortices have to be pinned by weaker pinning centers or trapped by the pinned neighbors with the help of inter-vortex repulsive magnetic interaction when the inter-vortex distance becomes comparable to the London penetration depth *λ*. The change from strong pining to weak collective pinning causes a rapid decrease of *J*_s_ and *U*_0_ (see Fig. 5(a)). Further increasing the magnetic field, most of the vortices are collectively pinned by weak pinning centers. However, since the inter-vortex distance is still much larger than the superconducting coherence length, vortex lines are still very stiff, vortex structure are only elastically deformed by random pinning or thermal fluctuations. At even higher magnetic fields (region III from *H*_on_(*T*) to *H*_cr._(*T*)), the inter-vortex distance becomes small and the overlap of vortex interaction becomes significant. Because the elastic constant *C*_44_ (tilt modulus) is usually much larger than *C*_66_ (shear modulus) for high temperature superconductor (high Ginzburg-Landau parameter κ)^48^, it is easier to shear vortex lines than to tilt them. Vortex lines can be easily bended, thus, better adjust to random pinning centers. Therefore, another mechanism trying to increase *J*_s_ and *U*_0_ through accumulating vortices is revealed. Those softened vortex lines are plastically deformed, and its number grows with increasing field, *i.e.*, an E–P crossover appears. However, *J*_s_ and *U*_0_ may still decrease with increasing field (in case of existence of E–P crossover while no SMP), since the total effect is determined by the competition of all the mechanisms mentioned above. Thus, the E–P crossover is not a sufficient condition for SMP. Absence of SMP is reported in FeSe crystal with innate superconductivity even though similar E–P crossover is observed^37^,^49^. SMP only appears when deformation of vortex lines wins the competition and this competition is related to many factors which we will discuss in the following paragraphs. About this physical scenario of SMP, a schematic diagram was provided in SI, Fig. S4. Accompanied with SMP, both *J*_s_ and *U*_0_ increase with field (see Fig. 5(a)). Note that vortices in both regions II and III creep mainly elastically and the boundary *H*_on_(*T*) is in fact not so definite. With *H*(*T*) increasing further (region IV from *H*_cr._(*T*) to *H*_irr_(*T*)), the plastic deformation becomes dominative and governs the vortex dynamics in a broad temperature and field range. In this case, the plastic deformation will gradually degrade in comparison with the decrease of pinning force and pinning energy of each pinning center with field increasing. Both *J*_*s*_ and *U*_0_ will decrease with field after reaching a maximum at *H*_sp_. Further increasing *H*(*T*) (region V from *H*_irr_(*T*) to *H*_c2_(*T*)), a melting phase transition from plastic vortex creep to unpinned vortex liquid is revealed.

SMP effect is affected by many factors. In the next, we will discuss some advantageous factors during this procedure of E–P crossover induced SMP. The predominant factor is the weak pinning centers from disorders. E–P crossover occurs when the pinning energy of additional effective pining centers due to the flux distortion can overcome the change of elastic energy of flux line. When the numbers of weak pinning center is increased, the E–P crossover becomes easier and SMP will move towards lower fields. The large set of weak pinning centers is the reason for that we can usually observe SMP in exotic-element-doped superconductors, such as iron-based Ba(Fe, Co)_2_As_2_. Irradiation is a useful technique to continuously induce effective pinning centers. With light irradiation to increase the number of weak pinning centers, SMP position should move towards lower fields. With heavy irradiation, strong pinning centers can be induced to cover the occurrence of SMP for the reason that the flux pinning will be dominated by the strong pinning center at the magnetic fields where the primal E–P crossover happens. Coverage of SMP by gradual increase of irradiation has been demonstrated in V_3_Si^50^.

Besides, the elastic property of the flux line is very important, including both the interaction between vortices and the strength of a single flux. The interaction between vortices is important for its close relation with the distance between vortices as well as the parameter penetration depth *λ* and coherence length *ξ*. As to the strength of a single flux, the two-dimensionality is very important. When the applied field is perpendicular to the surface plane of a layered superconductor, the rigidity of the single flux is dependent on the coupling between superconducting layers. If the coupling is weak and the single flux is soft, the flux line is easy to be distorted, and can translate from elastic to plastic vortices. That’s why SMP usually takes place in layered superconductors. It should be pointed out, the above mentioned factors is premised on good sample crystalline quality (uniform superconductivity). Generally, for polycrystalline sample, the *T*_c_ distribution is broad, which thereby causes no observation of SMP or only weak trace of SMP.

To the best of our knowledge, most of the SMP observed in single crystals in IBSs are accompanied with E–P crossover. Table 1 lists some magnetization relaxation studies on IBSs. The upper critical field *H*_c2_ anisotropy *γ* and the ratio between the *c*-axis coherence length *ξ*_*c*_ and the distance *d* between two adjacent FeAs/FeSe layers are also shown. To some extent, the ratio *ξ*_*c*_*/d* reflects the two-dimensionality of a superconductor. The lower value of *ξ*_*c*_*/d* indicates stronger two-dimensionality. Due to the similar layered structures, E–P crossover has been commonly demonstrated in different types of IBSs. In cases of Ca_1−*x*_Na_*x*_Fe_2_As_2_, FeSe, and H^+^-irradiated FeSe^37^,^42^,^49^, clear signal of E–P crossover has been witnessed, while no SMP occurs. This absence of SMP should be attributed to other factors including the numbers of weak pinning centers and also relatively large *ξ*_*c*_*/d* ratio. For the present 112-type iron-based superconductor, the anisotropy *γ* is moderate and the *ξ*_*c*_*/d* ratio is relatively low. Combined with the possible enhanced effective weak pinning centers from Co and/or La doping, E–P crossover and SMP emerge.

In summary, we have achieved large critical current density *J*_s_ ~ 2*10^6^ A/cm^2^ via Co-co-doping and observed the pronounced SMP phenomenon in the recently discovered 112-type superconductors. Our systematic magnetization study reveals a strong association between SMP and a crossover from elastic to plastic vortex creep crossover. The related factors in SMP occurrence have also been discussed in detail. Furthermore, even though the self-field *J*_*s*_ is relatively high in Co-co-doping 112 crystal, weak collective pinning is predominant in the present system. Applications of additive techniques such as irradiation and hydrostatic pressure should still be helpful for further *J*_*s*_ enhancement^49^,^51^.

## Methods

### Sample growth and basic characterizations

The Ca_0.8_La_0.2_Fe_1−*x*_Co_*x*_As_2_ single crystals were grown by self flux method. The starting materials with stoichiometric ratio of 0:15 : 0:9 :0:1 : 1:14(1 − *x*) 1:14*x* : 1 for CaO, Ca, La, FeAs, CoAs, and As were put into alumina crucibles and then sealed in vacuumed quartz tubes and heated in a high-temperature box furnace. After a low rate cooling process, crystals with typical size of 1–2 mm can be obtained. The compound composition was determined by muti-point energy dispersive x-ray spectral (EDS) measurements. The single crystal X-ray diffraction (XRD) data were collected through powder XRD measurement using a commercial Rigaku diffractometer with Cu K*α* radiation. Transport measurements were carried out by standard four-probe method on a physical properties measurement system (PPMS).

### Magnetization measurements

The magnetization data were collected using the VSM (vibrating sample magnetometer) option of PPMS. Both static and dynamic magnetization relaxations were performed. For static magnetization relaxation measurement, the detailed procedure is set as: (1) zero-field cooling (ZFC) the sample from temperature above *T*_*c*_ to the target temperature; (2) increasing field to 9 T with d*H* /d*t* = 191.3 Oe/s; (3) decreasing field to the target field with d*H* /d*t* = 100 Oe/s and starting continuous magnetization measurement typically to time *t* *>* 5400s; (4) repeating step (3) to accomplish the measurements for different target fields; (5) repeating steps (1–4) to finish the measurement for different temperatures. For dynamical relaxation measurement, *MH* curves with different field sweeping rates (d*H* /d*t* = 20, 100, 190 Oe/s) were recorded after a ZFC procedure to the target temperature. The raw data for static and dynamic relaxation measurements are shown in SI (Figs S2 and S3).

## Additional Information

**How to cite this article**: Zhou, W. *et al.* Second magnetization peak effect, vortex dynamics, and flux pinning in 112-type superconductor Ca_0.8_La_0.2_Fe_1-x_Co_x_As_2_. *Sci. Rep.* **6**, 22278; doi: 10.1038/srep22278 (2016).

## Supplementary Material

Supplementary Information

## Figures and Tables

**Table 1:** Several typical magnetization relaxation studies on IBSs.

Sample	*T*_c_	Results orconclusions	*γ* (*H*_c2_)	*ξ*_*c*_*/d*
Ba(Fe_0.93_Co_0.07_)_2_As_2_^7^ (**SMP**)	22 K	Collective to plastic creep crossover at *H*_sp_	1.5 ~ 2^46^	1.9
Ca(Fe_1-*x*_Co_*x*_)_2_As_2_ (*x* = 0.056)^39^ (**no SMP**)	19 K	Plastic creep rather than collective model	~1.3^52^	/
Ba_0.72_K_0.28_Fe_2_As_2_^12^ (**SMP**)	32.7 K	Single to collective at *H*_on_, collective to plastic at *H*_sp_	1 ~ 2^26^	4.4
Ca_0.25_Na_0.75_Fe_2_As_2_^42^ (**no SMP**)	33.4 K	Elastic to plastic near the upper end of power-law regime in *J*_c_(*H*) (*J*_c_ ∝ *H*^-α^, *α* = 0.55)	~2^53^	1.6 (estimated)
LiFeAs^14^ (**SMP**)	16.5 K	*U*_0_(*H*) peak occurs below *H*_sp_ → VL model	1.5 ~ 2.4^54^	2.7
FeTe_0.7_Se_0.3_^15^ (**SMP**)	10.6 K	Collective model, SMP was associated with vortex-vortex and vortex-defect interactions	1~2^27^	4.3
FeSe^37^ (**no SMP**)	9 K	Elastic to plastic creep crossover was observed	~1.8^55^	6.3
Ca_0.8_La_0.2_Fe_0.978_Co_0.022_As_2_ (present study)	37.8 K	Single to collective at *H*_s_, elastic to plastic near *H*_min._	2.7 ~ 4.2 (**SI**)	0.72 (estimated)
SmFeAsO_0.85_F_0.15_^8^ (polycrystalline)	46 K	3D flux line to 2D lattice	4 ~ 8^29^	0.21

*γ* is the upper critical field *H*_c2_ anisotropy ratio. *ξ*_*c*_ is *c*-axis coherence length and *d* is the distance between two adjacent FeAs/FeSe layers.
