# Supplementary material for: Second magnetization peak effect, vortex dynamics, and flux pinning in 112-type superconductor Ca0.8La0.2Fe1−xCoxAs2
Source: Sci Rep. 2016 Mar 7;6:22278. doi: 10.1038/srep22278 (PMC4780090; doi:10.1038/srep22278)
Supplement: Supplementary Information [file srep22278-s1.doc]

**Second magnetization peak effect, vortex dynamics, and flux pinning in** **112-type superconductor Ca**_0_*.*_8_**La**_0_*.*_2_**Fe**_1_*_−x_***Co***_x_***As**_2_

Wei Zhou, Xiangzhuo Xing, Wenjuan Wu, Haijun Zhao^*^ and Zhixiang Shi^†^

*Department of Physics and Key Laboratory of MEMS of the Ministry of Education, Southeast University, Nanjing 211189, China*

Email: ^*^haijunzhao @seu.edu.cn, ^†^[zxshi@seu.edu.cn](mailto:zxshi@seu.edu.cn)

1. **Critical current density**


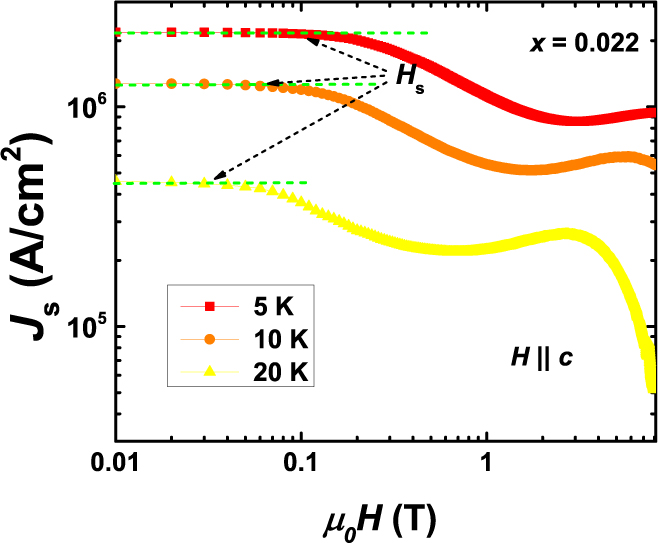


**Figure S1:** Log-log scale plot of the critical current density versus magnetic field. *H*_s_ is defined as the characteristic field for single-vortex region, which is determined by deviation from field-independent *J*_s_ region with increasing field.

1. **Static magnetization relaxation**

**Figure S2:** The typical time decay of magnetization plotted within a log-log scale in static relaxation measurements. Inset displays an example of interpolation formula (Eq. (2)) fitting of the raw magnetization data.

1. **Dynamic magnetization relaxation**

**Figure S3:** (a) Magnetic field dependence of magnetization measured under different field sweeping rate *dH*/*dt*. (b) Magnetization difference △*M* versus field sweeping rate *dH*/*dt*. △*M* is defined as the difference between the maximum and minimum magnetizations at the peak position *H*_sp_ and the dip position *H*_on_ on each *MH* curves.

1. **Physical vortex scenario for SMP**

**Figure S4:** The schematic diagrams for the vortex configurations under low, moderate, and high field. Based on our discussion, we suggest the sparse strong pinning centers and the dense weak pinning centers (represented by the larger and smaller dots, respectively) coexist in the sample. When the magnetic field strength is low, vortices are only pinned by strong pinning centers. With field increasing, weak pinning centers gradually become functional for more flux lines are induced. When the pinning energy of additional effective pining centers due to the flux distortion can overcome the change of elastic energy of the flux line, E-P transition occurs. For the reason that this vortex deformation benefits pinning, SMP takes place. Flux inside (outside) the sample is represented by the dashed (solid) line.

1. **Upper critical field anisotropy**

**Figure S5:** Temperature dependence of resistivity under different magnetic fields applied parallel or perpendicular to the crystal’s *c*-axis. As can be seen, the superconductivity is very robust against magnetic field for both field orientations, indicating high sample quality. The upper critical field *H*_c2_ in the phase diagram was extracted from this figure by criterion of 90% *ρ_n_*. Here, *ρ_n_* is normal state resistivity. The *H*_c2_ anisotropy *γ* is estimated around 2.7-4.2, which verifies the sample as a good candidate for link study of vortex physics between iron pnictides and cuprates
